# Novel Heterotypic Rox Sites for Combinatorial Dre Recombination Strategies

**DOI:** 10.1534/g3.115.025841

**Published:** 2015-12-29

**Authors:** Katherine Chuang, Eileen Nguyen, Yuri Sergeev, Tudor C. Badea

**Affiliations:** Retinal Circuits Development and Genetics Unit, National Eye Institute, National Institutes of Health, Bethesda, Maryland 20892

**Keywords:** site specific recombination, Cre recombination, Dre recombinase, gene targeting

## Abstract

Site-specific recombinases (SSRs) such as Cre are widely used in gene targeting and genetic approaches for cell labeling and manipulation. They mediate DNA strand exchange between two DNA molecules at dedicated recognition sites. Precise understanding of the Cre recombination mechanism, including the role of individual base pairs in its loxP target site, guided the generation of mutant lox sites that specifically recombine with themselves but not with the wild type loxP. This has led to the development of a variety of combinatorial Cre-dependent genetic strategies, such as multicolor reporters, irreversible inversions, or recombination-mediated cassette exchange. Dre, a Cre-related phage integrase that recognizes roxP sites, does not cross-react with the Cre-loxP system, but has similar recombination efficiency. We have previously described intersectional genetic strategies combining Dre and Cre. We now report a mutagenesis screen aimed at identifying roxP base pairs critical for self-recognition. We describe several rox variant sites that are incompatible with roxP, but are able to efficiently recombine with themselves in either purified systems or bacterial and eukaryotic tissue culture systems. These newly identified rox sites are not recognized by Cre, thus enabling potential combinatorial strategies involving Cre, Dre, and target loci including multiple loxP and roxP variants.

SSRs such as Cre, Flp, and phiC31 are used extensively in genetic model organisms to direct tissue and/or time-dependent genetic manipulations (Sauer and Henderson 1988; Grindley *et al.* 2006; Bischof and Basler 2008; Birling *et al.* 2009; Jefferis and Livet 2012; Turan *et al.* 2013; Jensen and Dymecki 2014). Typical application involves the expression of a recombinase (*e.g.*, Cre) in a particular subset of cells, inducing a recombination event at its target sites (*e.g.*, loxP sites) resulting in gene ablation or induction of a reporter or effector gene. As the resolution of addressed biological questions increases, so does the requirement for restricting our genetic manipulations to specific cell types and time windows. Spatially and/or temporally restricted manipulation is typically achieved by intersecting the expression patterns of existent or engineered gene regulatory elements that control the expression of the recombinase and its target. The target will be recombined in all tissues expressing the Cre, however, only the subset that also expresses the targeted gene or reporter will exhibit a phenotype, cell labeling, or other desired manipulation. Modern gene expression analyses reveal broadly overlapping gene expression profiles, and in only a few instances can unique cell types be suitably approached by intersecting the expression domains of two genes. This issue is particularly critical in the nervous system, where a multitude of cell types are present (Jefferis and Livet 2012). Therefore, intersectional strategies using multiple recombinases, genetic loci, and/or local vector delivery are of great interest (Fenno *et al.* 2014; Madisen *et al.* 2015).

The Cre-loxP recombination system is by far the most developed of the genetic manipulation toolbox, benefitting from a variety of mouse lines expressing Cre or Cre-dependent targets (Nagy *et al.* 2009) and flexibility of strategies used to generate tightly regulated or combinatorially recombined target sites (Schnutgen *et al.* 2003; Livet *et al.* 2007; Atasoy *et al.* 2008). These targeting methodologies have been made possible by our extensive knowledge of the Cre-loxP recombination mechanism (Gopaul and Van Duyne 1999). The loxP target site consists of two 13 bp inverted repeats, flanking an asymmetric 8 bp spacer (Figure 1A, horizontal arrows) (Hoess and Abremski 1984; Hoess *et al.* 1986; Guo *et al.* 1997). Cre recombination involves the formation of a complex containing two loxP sites and four Cre proteins. During recombination, each inverted repeat of the two lox sites is bound specifically by one Cre protein. The spacer regions undergo single strand breaks, followed by strand exchange, strand migration and formation of a Holiday junction, a second cleavage and strand exchange, and finally the resolving of the Cre-loxP complex (Guo *et al.* 1997; Gopaul and Van Duyne 1999; Grindley *et al.* 2006). The asymmetric spacer base pairs are involved in the catalytic reaction, and ensure the unidirectionality of the Cre-loxP reaction. If the two loxP sites present on the same DNA segment have identical orientations, Cre recombination results in an excision reaction, while loxP sites with opposing orientation produce inversion reactions. loxP strand breaks occur between spacer positions 1–2 on the top strand and 7–8 on the bottom strand (Figure 1A, vertical arrows) (Hoess and Abremski 1985; Guo *et al.* 1997). Several screens have been performed to explore the influence of the 8 bp spacer sequence on recombination efficiency and specificity (Lee and Saito 1998; Siegel *et al.* 2001; Langer *et al.* 2002; Missirlis *et al.* 2006; Jung *et al.* 2007). Systematic base substitution analysis showed that single mutations of the loxP spacer at position 7, or double mutants involving position 7 plus any of positions 2–5 (*e.g.*, lox2272), yield mutant spacers that do not recombine with the wild type (WT), but do recombine efficiently with themselves. It is proposed that mutations of positions 6 and 7 (Figure 1A) interfere with the initial strand exchange and therefore recombination initiation, while mutations at positions 2 through 5 arrest the loxP recombination in the intermediate states (Lee and Saito 1998; Gopaul and Van Duyne 1999). The two alternative lox site configurations (direct or reversed) combined with lox sites (lox511 and lox 2272) that do not recombine with loxP, but self-recombine efficiently (Lee and Saito 1998; Siegel *et al.* 2001) are the base for the design of combined inversion–excision or alternative excision strategies, such as FLEX and Brainbow (Jefferis and Livet 2012).

**Figure 1:**
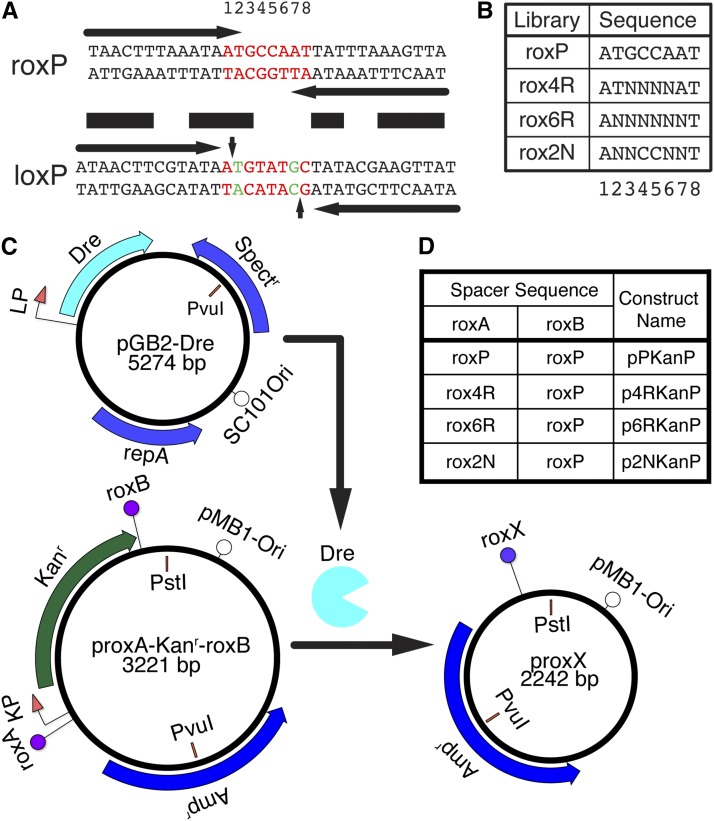
Random nucleotide libraries targeted at the roxP spacer region. (A) Comparison of roxP (top) and loxP (bottom) wild type sites. Horizontal arrows mark the inverted repeats for each target site and the black horizontal bars label identical base pairs. Regions highlighted in red correspond to the spacer region of the loxP site and the equivalent base pairs on the roxP site (numbered 1–8). The vertical arrowheads on the loxP sequence mark the sites of catalytic attack and strand break during Cre recombination. Mutations at positions 2 and 7 in the loxP spacer, highlighted in green, are used in the lox2272 mutant. (B) Libraries carrying random nucleotides indicated by N’s inserted at positions 3–6 (rox4R), 2–7 (rox6R) and 2, 3, 6, and 7 (rox2N) in the spacer region of roxP. (C) Plasmids and recombination test used in this paper. pGB2-Dre contains a replication cassette consisting of the repA protein and SC101 origin of replication (ensuring 1–5 copies/cell), a spectinomycin resistance gene (Spc^r^), and a Dre open reading frame driven by a lac promoter (LP). proxA-Kan^r^-roxB contains a pMB1 origin (generating >75 copies/cell), an ampicillin resistance gene, and a kanamycin (Kan^r^) resistance cassette, flanked by two rox sites arranged in direct orientation, roxA and roxB. If both rox sites are occupied by wild type roxP sequences (pPKanP vector), Dre recombination results in deletion of the intervening fragment containing the Kan^r^ element. In the unrecombined plasmid, *Pvu*I + *Pst*I digestion results in 1.8 kb and 1.4 kb fragments, while after Dre recombination and Kan^r^ removal the 1.8 kb fragment is reduced to 0.8 kb, while the 1.4 kb fragment (containing the pMB1-Ori) is unaffected. (D) Wild type roxP sites were inserted at the roxB position, while the mutant rox sites carrying the random nucleotide libraries shown in B were cloned into the roxA position, generating the p4RKanP, p6RKanP, and p2NKanP vectors.

Among many recombinases recently added to the toolbox of genetic manipulations, Dre has several features that make it particularly interesting (Sauer and McDermott 2004; Anastassiadis *et al.* 2009). It is a close homolog of Cre, but has similar recombination efficiency and specificity for its own target sites, while exhibiting no cross-reactivity with the other well established recombinases, Cre and Flp. Several mouse and zebrafish models have been developed and validated demonstrating both the feasibility of Dre recombination in genetic manipulations and its use in combinatorial approaches with other recombinases (Anastassiadis *et al.* 2009; Park and Leach 2013; Fenno *et al.* 2014; Sajgo *et al.* 2014; Madisen *et al.* 2015). Some of these studies have reported a moderate level of cross-talk between Dre and Cre, especially in the context of viral delivery of the recombinases and targets. Cre and Dre protein sequences are 40% homologous and all Cre active site amino acids (Arg173, His289, Arg292, Trp315, and Tyr324) are conserved in Dre (Arg175, His290, Arg293, Trp316, and Tyr325). Furthermore, there is partial conservation of amino acids and base pairs involved in the interface between Cre and the loxP inverted repeat (Hoess and Abremski 1984, 1985; Hoess *et al.* 1990; Guo *et al.* 1997; Sauer and McDermott 2004). We therefore speculated that the mechanisms of Dre and Cre might be similar and that the pattern of protein to DNA interaction over the inverted repeat and spacer area are conserved. However, the inverted repeats of the roxP site are 14 bp long, while the asymmetric internal spacer is 4 bp long (Figure 1A), (Sauer and McDermott 2004). This precludes a simple alignment with the loxP site. Moreover, the regions of identity between the two sites are mostly confined to the inverted repeats, and reach into the first 3 bp of the loxP spacer on only one side (base pairs 1–3, ATG, Figure 1A). Thus, it is not clear which base pairs of the roxP site might be the functional equivalents of the critical base pairs 2 and 7 in loxP.

We therefore designed a mutation selection screen to identify roxP variants that are not compatible with the WT roxP site but preserve their ability to self-recombine. We have identified several such mutant spacers, and demonstrate their ability to function in a specific and efficient manner in prokaryotic and eukaryotic cell contexts, and analyzed their self-recombination efficiency in a purified system. This recombination specificity and efficiency allows for the development of a tight inversion–excision strategy, which we call FREX.

## Materials and Methods

### Vector design and construction

**Figure 2:**
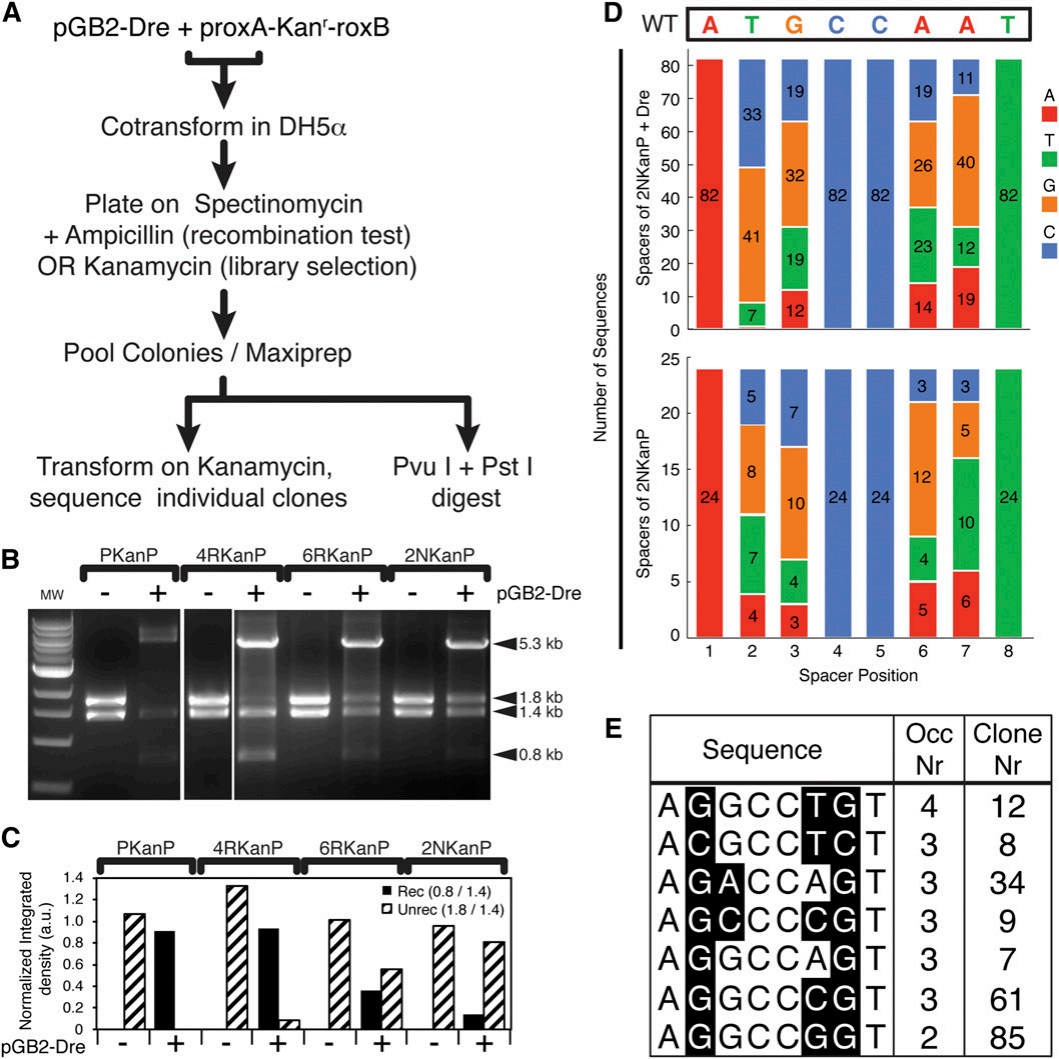
Screen strategy and results for identification of mutant rox sites. (A) Strategy used for recombination test (for p4RKanP, p6RKanP, or p2NKanP libraries) and for library screening (p2NKanP library). For details, see also *Material and Methods*. (B) *Pvu*I + *Pst*I restriction digest analysis of pooled target vectors before (−) and after (+) exposure to Dre recombination. Linearized pGB2-Dre can be seen in the (+) lanes (5.3 kb). Target vectors show the unrecombined restriction pattern (1.4 kb + 1.8 kb) before pGB2-Dre cotransformation (− lanes). After cotransformation (+ lanes), various degrees of recombination (0.8 kb band) can be seen. (C) Densitometric analysis of recombination efficiency of libraries and wild type control. (D) Nucleotide distributions at positions 1–8 in 82 individual p2NKanP minipreps sequenced after cotransformation with pGB2-Dre, and Kan selection (top) and 24 control p2NKanP minipreps from the unrecombined library (bottom). Original roxP sequence marked as wild type (WT) is indicated at the top. Positions 1, 4, 5, and 8 were not mutated in the rox2N spacer library. Numbers in the colored bars represent number of spacer sequences having the indicated nucleotide at the respective position. Nucleotide distributions were not significantly different between Dre-treated and untreated libraries at positions 3 (χ^2^ = 0.73, *P* = 0.87) and 6 (χ^2^ = 3.84, *P* = 0.28). However, after Dre selection, position 2 shows a highly significant bias toward G and C (χ^2^ = 18.44, *P* = 0.000357), and position 7 a significant bias toward G (χ^2^ = 9.99, *P* = 0.0186). (E) Most frequent spacers recovered from the screen. Nucleotides differing from consensus are shown in black. Occ. Nr. represents the number of times that specific sequence was observed. Clone number is the representative clone used for further studies.

**Figure 3:**
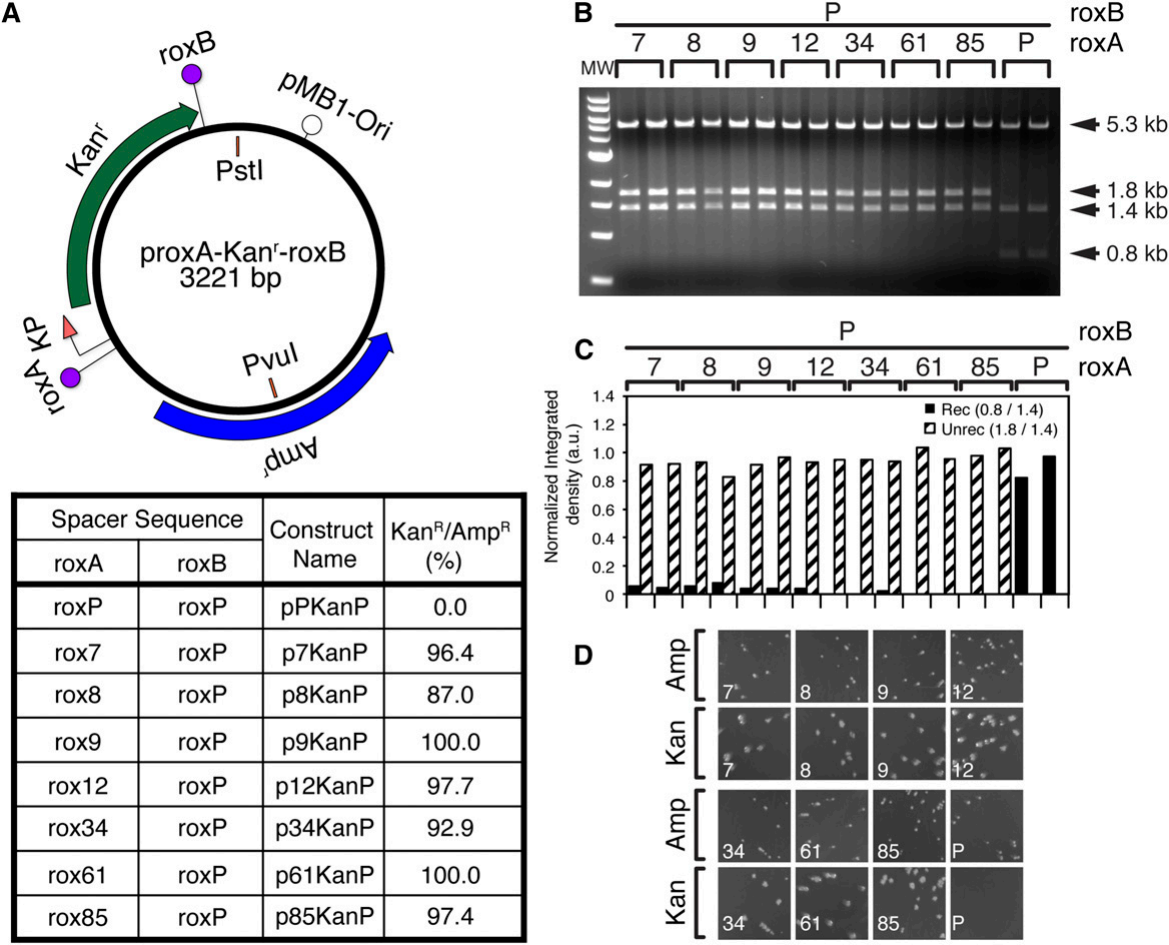
Identified rox mutants are indeed unable to recombine with the wild type. Seven of the identified mutant spacers (A) were tested for the ability to recombine with the wild type roxP site, by cotransformation with pGB2-Dre in DH5α. Transformations were plated on ampicillin^+^ spectinomycin^+^, and two minipreps for each clone were amplified and diagnosed by restriction digest with *Pvu*I + *Pst*I (B, C). In addition, both clones were retransformed into DH5α plated on ampicillin plates, and replica plated using a velvet replicator onto kanamycin plates (D). (A) Schematic of the generic recombination target construct (top), and naming convention for the individual mutant constructs (bottom table). The roxB site is always roxP. The roxA site is either a wild type rox site (pPKanP) or one of the seven mutant spacers (Figure 2E). (B) Digest with *Pvu*I + *Pst*I. The 5.3 kb band represents the linearized pGB2-Dre. Both isolates of all seven mutant clones failed to recombine (1.8 + 1.4 kb bands), while the wild type isolates fully recombined (1.4 + 0.8 kb fragments). (C) Densitometric analysis confirms minimal recombination for mutants 7–9, and essentially no recombination for mutants 12–85. (D) Replica plating experiments for one of the two isolates for each clone, as well as the wild type control. For each clone, the number is indicated in the bottom left corner. Top rows are initial ampicillin plates, and bottom rows replicates onto kanamycin plates. The ratio of colonies surviving on kanamycin *vs.* ampicillin plates is shown as percent in A, bottom table. Images of full plates are provided in Figure S2.

**Figure 4:**
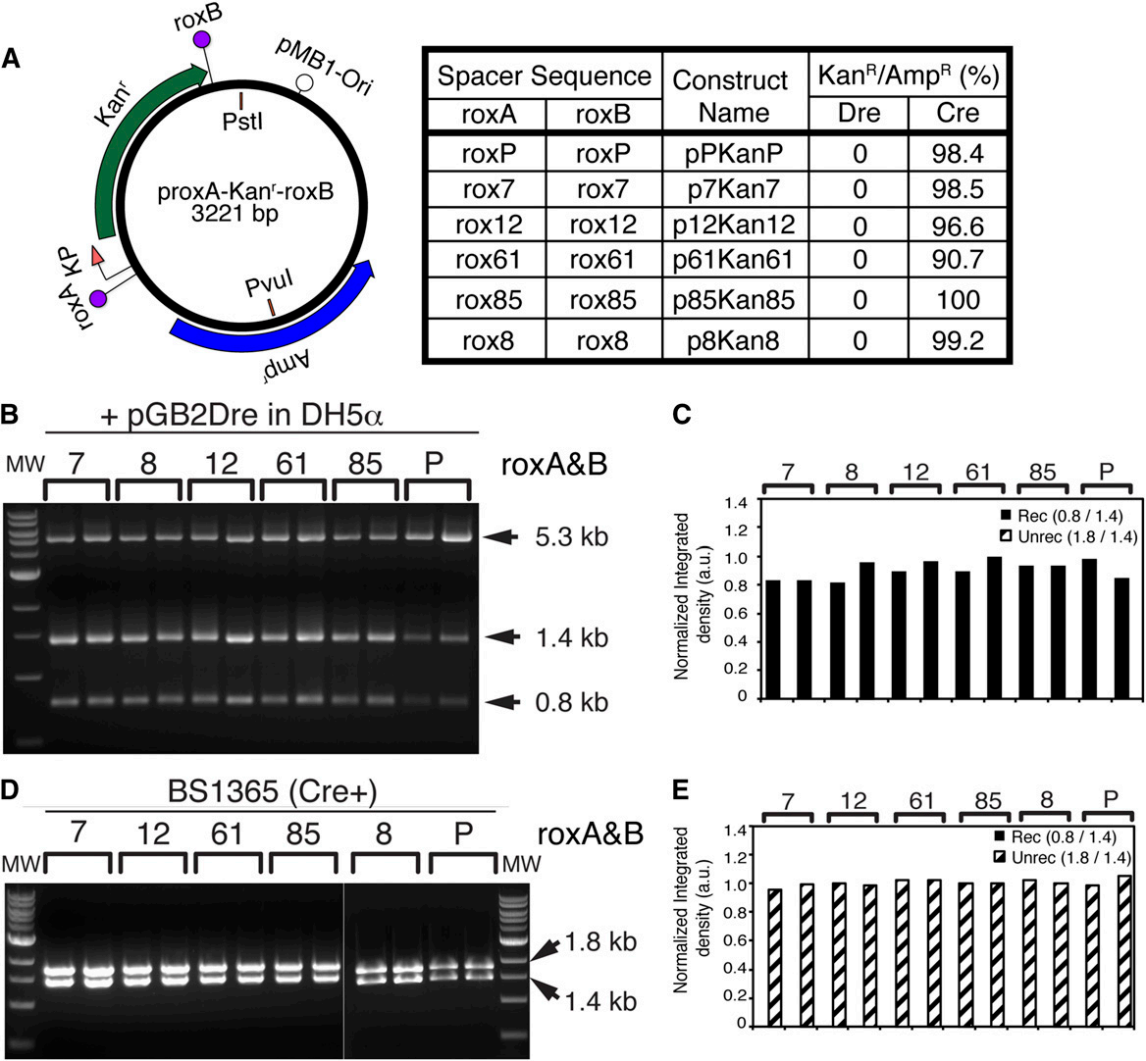
Rox mutants rox7, rox8, rox12, rox61, and rox85 recombine with themselves in the presence of Dre but not Cre recombinase. (A) Rox sites with spacers identical to clones 7, 8, 12, 61, and 85 (Figure 2E) were cloned at both the roxA and roxB positions in direct orientation, generating the p7Kan7, p8Kan8, p12Kan12, p61Kan61, and p85Kan85 constructs. Each construct, as well as the pPKanP positive control, were then exposed to Dre recombinase by the same cotransformation protocol outlined in Figure 3. Two separate isolates for each clone were then tested by *Pvu*I + *Pst*I restriction digestion (B, C) and replica plating (Figure S3, A, B, C, and F). All rox sites tested showed complete recombination (1.4 + 0.8 kb fragments) when exposed to Dre recombinase, and essentially no surviving colonies when replica plated from ampicillin to kanamycin plates (Figure S3A, and table in A, right hand column). Quantitations of B are shown in C. To test the sensitivity of the wild type roxP and mutants to Cre recombination, pPKanP and p7Kan7, p8Kan8, p12Kan12, p61Kan61, and p85Kan85 mutants were transformed in the BS1365 strain, constitutively expressing Cre. Transformations were plated on kanamycin and ampicillin, and two individual colonies from each clone were amplified and analyzed by *Pvu*I + *Pst*I restriction digestion (D, E). In addition, DNA from the two colonies was retransformed in DH5α, plated on ampicillin, and colonies replica plated onto kanamycin plates using a velvet replicator (Figure S3B). All tested constructs show the 1.8 and 1.4 kb fragments characteristic for lack of recombination (D) and resistance to kanamycin in the replica platting assay (Figure S3B and table in A, right hand column). Note that the BS1365 strain carries the Cre recombinase on a F’ element that also contains a kanamycin resistance gene. Therefore, kanamycin does not select against rox target vector recombinants in the initial BS1365 cotransformation step.

Bacterial Dre expression and target library selection constructs were built in vectors with distinct replication origins, allowing for cotransformation in bacteria. pGB2-Dre was generated by inserting a *lac* promoter – Dre cassette into pGB2 between *Eco*RI and *Hin*dIII sites (Figure 1C) (Churchward *et al.* 1984). *Dre dependent roxP recombination target plasmids and derivatives:* a kanamycin resistance cassette (Kan^r^) was PCR amplified from pBBRBB-eGFP (Vick *et al.* 2011) and subcloned between *Xba*I and *Not*I into pUCBB-eGFP (Vick *et al.* 2011), replacing the eGFP expression cassette. A WT roxP site (5′-TAACTTTAAATAATGCCAATTATTTAAAGTTA-3′) was inserted between *Not*I and *Xho*I, downstream of the Kan^r^ gene (pKan-roxP). Next, a WT roxP site was inserted between *Eco*RI and *Nhe*I/*Xba*I sites upstream of the Kan^r^ cassette, to generate pPKanP (Figure 1C). Random oligonucleotide insertion libraries (Figure 1B): rox4R (5′-TAACTTTAAATAATNNNNATTATTTAAAGTTA-3′), rox6R (5′-TAACTTTAAATAANNNNNNTTATTTAAAGTTA-3′), rox2N (5′-TAACTTTAAATAANNCCNNTTATTTAAAGTTA-3′) were custom ordered to be commercially built with hand-mixed bases (IDT) and amplified by PCR, adapting *Eco*RI and *Nhe*I sites at the ends. They were inserted in pKan-roxP upstream of the kanamycin promoter, between *Eco*RI and *Xba*I generating the p4RKanP, p6RKanP, or p2NKanP vector libraries (Figure 1D). Diversity of the libraries was confirmed by sequencing 12 individual clones and comparing the base pair distributions at the mutated sites. For p2NKanP, a total of 24 minipreps were sequenced, and the results are summarized in Figure 2D, bottom panel. Plasmids p7KanP, p8KanP, p9KanP, p12KanP, p34KanP, p61KanP, and p85KanP (Figure 2E and Figure 3A) are clones identified during the screen. Vectors p7Kan7, p8Kan8, p12Kan12, p61Kan61, and p85Kan85 (Figure 4A) were generated by replacing the WT roxP downstream of the Kan^r^ gene in p7KanP, p8KanP, p12KanP, p61KanP, and p85KanP, respectively, with rox sites carrying the mutated spacer sequences rox7 (AgGCCAgT), rox8 (AcGCCtcT), rox12 (AgGCCtgT), rox61 (AgGCCcgT), and rox85 (AgGCCggT), between *Not*I and *Xho*I. *Eukaryotic roxP recombination targets*: An eGFP-bGH-PA (bovine growth hormone polyadenylation signal) cassette was inserted in pcDNA3.1(-) (Invitrogen) between *Eco*RI-*Hin*dIII sites. The Kan^r^ cassette in vectors pPKanP, p12Kan12, p12KanP, p85Kan85, and p85KanP was replaced with a mCherry-3xSTOP (3 × SV40 polyA transcription termination site) inserted between *Not*I and *Pvu*II restriction sites, thus being flanked by the respective roxA and roxB sites of each vector. The orientation of the mCherry-3xSTOP is reversed compared to the orientation of the Kan^r^ cassette. Then, the rox-mCherry-3xSTOP-rox cassettes from each of the five vectors: pPCherryP, p12Cherry12, p12CherryP, p85Cherry85, and p85CherryP, were subcloned into the pcDNA3.1(-)-EGFP-bGH-PA vector between *Xho*I and *Eco*RI sites, resulting in the final eukaryotic target vectors: proxP-R-roxP-G, prox12-R-rox12-G, proxP-R-rox12-G, prox85-R-roxP-85, and proxP-R-rox85-G (Figure 6A).

**Figure 5:**
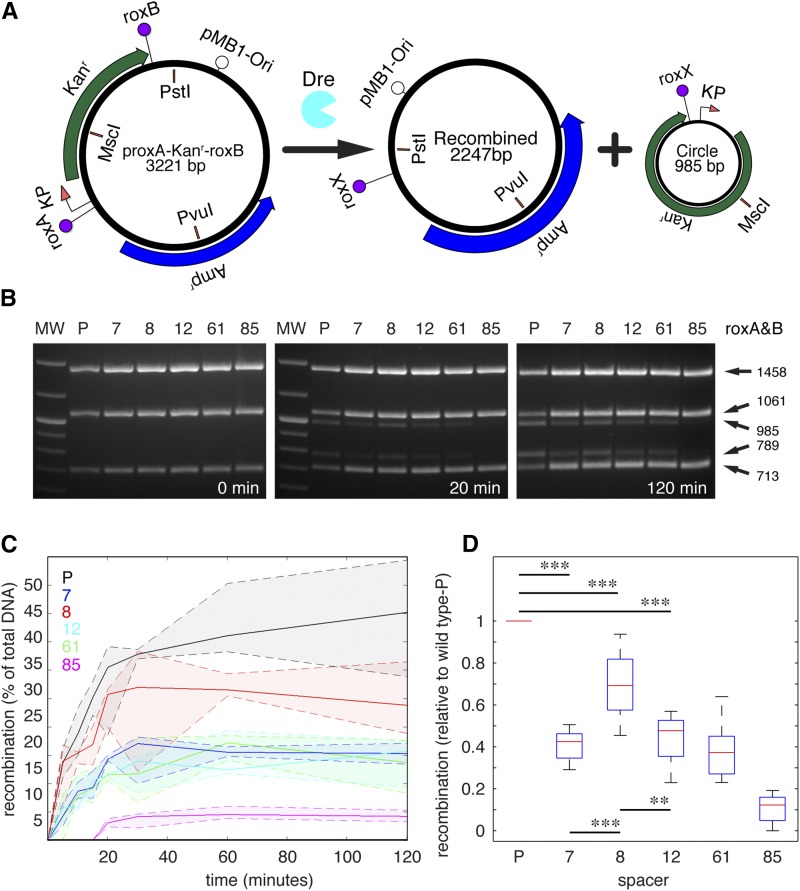
Self-recombination efficiencies of roxP and novel sites tested in purified system. (A) Diagram of *in vitro* recombination reaction. proxA-Kan^r^-roxB DNA is exposed to affinity-purified Dre, resulting in a recombined plasmid and a circle. Digestion with *Pvu*I + *Pst*I + *Msc*I results in a common *Pst*I-*Pvu*I 1458 bp fragment spanning the pMB1 origin and part of the Amp^r^ and two alternative, recombination-dependent sets of fragments. Without recombination, a *Msc*I–*Pst*I 713 bp fragment spanning the roxB site and a *Pvu*I–*Msc*I 1061 bp fragment spanning the roxA can be detected (see for instance the 0 min timepoint in B). After recombination, a *Pst*I–*Pvu*I fragment of 789 bp spanning the roxX site is observed in the remaining vector, and the 985 recombination circle is linearized by *Msc*I. Without *Msc*I digestion, the recombination circle migrates at about 600 bp (not shown). (B) Representative gels for the 0, 20, and 120 min time points reveal gradually accumulating recombination products. Lanes represent either marker (MW), or the prox-Kan^r^-rox vectors carrying either wild type (P) or spacers 7, 8, 12, 61, and 85 at both roxA and roxB sites (Figure 4A). Note that samples for the 20 and 120 min time points are taken from the same gel. (C) Densitometric analysis revealing percent recombination over the 120 min timecourse for wild type and all five mutant spacers. Solid lines represent medians, and dotted lines 25^th^ and 75^th^ quartiles for each set of samples (n = 6 data points/spacer and time point). (D) Box plots for the 60 min time point. Recombination efficiencies for each mutant spacer are shown as ratios to the wild type (P) from the same experiment (n = 10 data points/spacer). (Kolmogorov–Smirnov two sided test, significance levels ** *P* < 0.005, *** *P* < 0.001.)

FREX cassette vector (pAAVFREX-eGFp-mCherry): Synthetic oligos containing the rox12 and roxP WT arranged in tandem in direct and reverse orientation surrounding a multicloning site were assembled in pBSKS (pBSKS-FREX). The FREX cassette was then cloned using a *Bam*HI to *Eco*RI into an AAV expression vector containing AAV2 ITRs, a ubiquitously expressing chicken β-actin enhancer CMV promoter fusion (CAG) (Atasoy *et al.* 2008), followed by a WPRE (a posttranscriptional regulatory element derived from a woodchuck hepatitis virus (Zufferey *et al.* 1999) and bovine Growth Hormone polyadenylation signal, resulting in the pAAVCAGFREX vector. An eGFP cDNA in direct orientation, and a mCherry-SV40 polyA transcription STOP cassette in reverse orientation relative to the promoter, were cloned into the multicloning site (pAAV-FREX-eGFP-mCherry, Figure 7). The SV40 polyA sequence contains transcriptional stop and polyadenylation signals in both orientations, and thus will also serve as a transcriptional terminator after inversion. The control vector pAAVPTPY contains a PSD95-TFP-P2A-GAP43-eYFP expression cassette cloned in inverted orientation in the pAAVFLEX vector containing inverted tandem copies of loxP and lox2272 (Atasoy *et al.* 2008) (B. Wu and T. C. Badea, unpublished results).

### Bacterial recombination assay and library screening

Bacterial strains (*Escherichia coli*) were DH5α for Dre recombination tests and cloning, and BS1365 (F’Kan, recA1, endA1, λ imm434 x1-Cre), a generous gift from Dr. Brian Sauer, for testing Cre–roxP interactions. For the *Library Dre recombination test*, pGB2-Dre (Spc^r^) was cotransformed into DH5α bacteria with either the WT control plasmid pPKanP or one of the proxA-Kan^r^-roxB (Kan^r^, Amp^r^) target libraries, and transformants were plated onto spectinomycin^+^ ampicillin^+^ selection plates. Colonies from 10–20 plates were scraped off the plates, allowed to grow under selection, and then submitted to large scale DNA extraction. An aliquot of the resulting DNA was digested with *Pvu*I + *Pst*I. *Library screening* for the rox2N library was performed essentially as described for the recombination assay, except that the cotransformation was plated on spectinomycin + kanamycin, and pooled colonies were further amplified on kanamycin, to ensure stringent selection for unrecombined target plasmids. Preliminary tests showed that Spc^r^ does not convey kanamycin resistance nor does Kan^r^ spectinomycin resistance (data not shown). About 50 ng of plasmid DNA was then retransformed in DH5α and selected on kanamycin, and plasmid DNA was isolated from individual colonies and tested for recombination by restriction digest with *Pvu*I + *Pst*I. Mutated rox sites were sequenced for 92 colonies. Ten of the sequenced clones had missing or mutated direct inverted repeats, outside the spacer region, and were discarded from further analysis. For recombination assays for clone confirmation and testing mutant self-recombination, the target plasmids were cotransformed with pGB2-Dre onto spectinomycin^+^ ampicillin^+^ selection. For each target, two isolated colonies were miniprepped, and DNA digested with *Pvu*I and *Pst*I as above, or retransformed on ampicillin plates. Colonies from the ampicillin plates were replicated onto kanamycin plates using sterilized velvet replicators.

### Recombination in purified system

The DNA substrates were the prox-Kan^r^-rox vectors series used for checking homotypic recognition, p7Kan7, p8Kan8, p12Kan12, p61Kan61, and p85Kan85, along with the pPKanP control vector. The Dre protein was produced as follows. The Dre sequence was subcloned into the pET-28a vector, which carries an N-terminal histidine tag. Cultures growing in log phase were subjected to IPTG induction for 2 hr before cells were harvested. The pellet was resuspended in 1 mM PMSF in PBS and sonicated for 12 cycles of 10 sec on, 5 sec off at 30 W. Expression of the His-tagged Dre protein (MW = 42,343) was tested by colloidal blue (Simply Blue, Invitrogen) staining of SDS-PAGE. The Dre protein was purified using a HiTrap Nickel column, followed by dialysis against storage buffer overnight at 4°. Dre protein yield and purity were determined by quantifying the total protein concentration using a BCA Assay (Pierce BCA protein assay) combined with densitometric evaluation of SDS-PAGE gels stained with coloidal blue. The purified protein was stored at –80° in 10 mM Tris-HCl, 150 mM NaCl, and 0.5 mM EDTA. For the recombination reaction, the protein was kept in storage buffer containing 40% glycerol at –20°. The buffer optimized for the recombination reaction contained: 50 mM Tris, 100 μg/ml BSA, 10 mM MgCl, 50 mM NaCl, and 1 mM DTT. Vectors were linearized with *Pst*I for 1 hr at 37°, followed by heat inactivation (20 min at 70°), and then DNA (∼0.25 mmol) was incubated with Dre protein (∼2 mmol) at 37° and the recombination reactions were terminated at given time points by heat-inactivation of the Dre protein at 70°. The DNA was then further digested with *Pvu*I and *Msc*I to generate diagnostic fragments allowing the differentiation of unrecombined from recombined products. Diagnostic digests were then resolved on a 1.5% agarose gel stained with ethidium bromide, and band densitometric analysis was performed using ImageJ (Wayne Rasband, NIH, http://imagej.nih.gov/ij).

### HEK293 recombination assay

**Figure 6:**
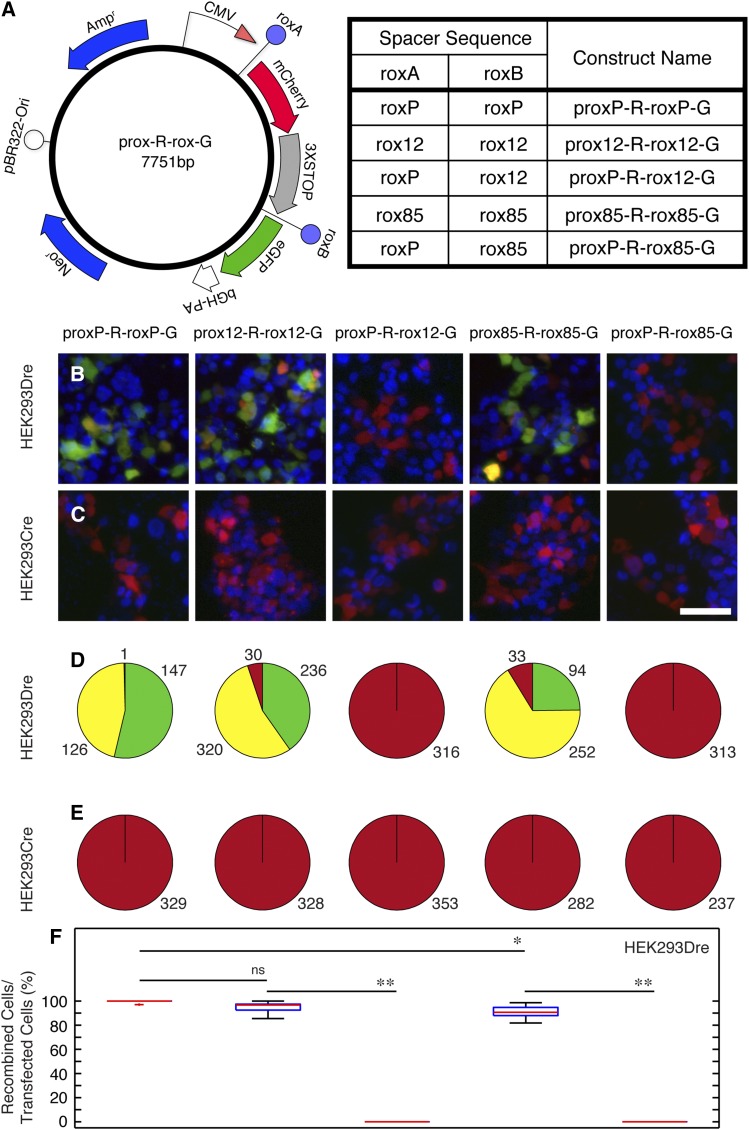
Rox mutants rox12 and rox85 recombine with themselves but not with roxP in eukaryotic cells. (A) In the prox-R-rox-G eukaryotic expression vector, a CMV promoter is driving transcription of mCherry before Dre recombination between the roxA and roxB sites, and of eGFP after recombination. rox12 and rox85 mutants or roxP wild type control were placed at both roxA and roxB sites to generate prox12-R-rox12-G, prox85-R-roxP-85 and proxP-R-roxP-G vectors, suitable for testing the ability of each rox site to recombine with itself. In addition, rox12 or rox85 mutants were cloned at the roxB site in combination with a roxP at the roxA site, to generate proxP-R-rox12-G and proxP-R-rox85-G, to test the compatibility between rox mutants and roxP wild type. (B, C) HEK293 cells stably expressing either the Dre (B) or Cre (C) recombinase were transfected with the constructs described in A. (D, E) Numbers in pie charts indicate the sums for red (mCherry only), green (eGFP only), and yellow (double positive) cells over six individual 20 × fields derived from two distinct transfected coverslips for each of the experimental conditions in B and C. No eGFP positive cells were seen in HEK293Dre cells transfected with proxP-R-rox12-G and proxP-R-rox85-G (B, D, columns 3 and 5) or HEK293Cre cells transfected with either one of the five constructs (C, E). (F) Box-whisker plots quantitating the percent of recombination positive cells in the HEK293Dre experiments. The percentage represents 100 × (eGFP^+^ + eGFP^+^mCherry^+^)/(eGFP^+^ + eGFP^+^mCherry^+^ + mCherry^+^). Statistical significance was determined by student *t*-test and Kolmogorov–Smirnov test (n.s. = *P* > 0.05, * = *P* < 0.05, ** = *P* < 0.005). Scale bar in C = 50 µm.

**Figure 7:**
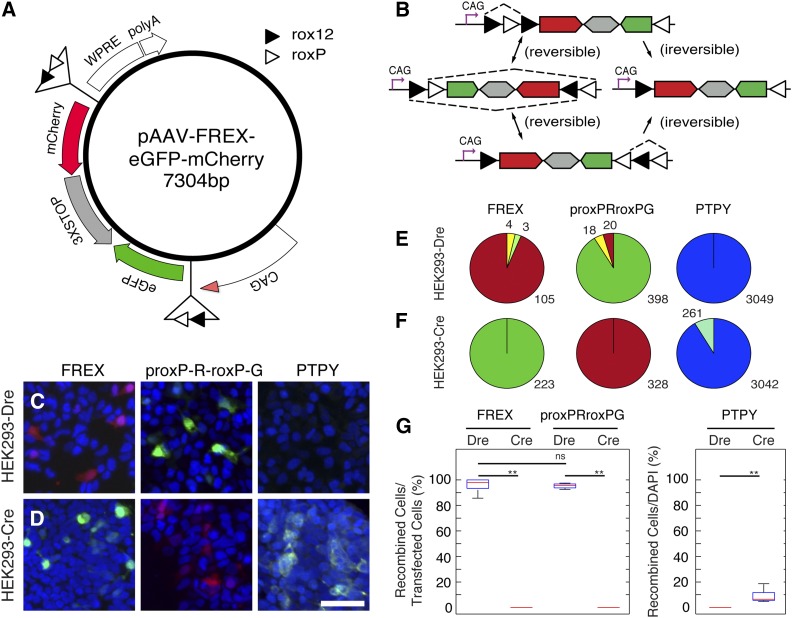
Efficient Dre recombination using an inversion–excision cassette (FREX) based on rox12 and roxP. (A) The pAAV-FREX-eGFP-mCherry eukaryotic viral expression vector contains a CAG promoter driving an expression cassette flanked by tandem rox12-roxP sites. The cassette contains eGFP and mCherry cDNAs placed in reverse orientation to each other, and separated by a triple repeat of a bidirectional SV40 transcription stop/polyadenylation signal. WPRE and bGH elements ensure mRNA stability and processing and AAV2 ITRs (not shown) allow for viral packaging. (B) Dre induces two alternative inversion reactions (double arrows) followed by irreversible excision (single arrow) reactions. The CAG promoter drives transcription of eGFP before, and of mCherry after, Dre recombination. HEK293-Dre (C) or HEK293-Cre (D) cells were transfected with FREX (column 1), prox-R-rox-G (column 2), or pAAVPTPY, a FLEX-based Cre reporter plasmid (column 3). (E, F) Numbers in pie charts for columns 1 and 2 indicate the sums for red (mCherry only), green (eGFP only), and yellow (double positive) cells. Results from cells transfected with pAAVPTPY (cyan) are shown in column 3 (E, F), comparing recombined cells to the total number of cells (DAPI, blue). Only Hek293Cre cells recombined the PTPY construct. (G) Box-whisker plots quantitating the percent of recombination positive cells. The percentage represents 100 × (mCherry^+^ + eGFP^+^mCherry^+^)/(eGFP^+^ + eGFP^+^mCherry^+^ + mCherry^+^) for FREX transfections or 100 × (eGFP^+^ + eGFP^+^mCherry^+^)/(eGFP^+^ + eGFP^+^mCherry^+^ + mCherry^+^) for pRoxP-R-roxP-G transfections. Recombination of PTPY is represented by 100 × Cyan^+^/DAPI^+^. Recombination efficiency was not statistically significant between the FREX and pRoxP-R-roxP-G in Dre-expressing cells. Statistical significance was determined with student *t*-test and Kolmogorov–Smirnov test (n.s. = *P* > 0.05, ** = *P* < 0.005). Scale bar in C = 50 μm.

HEK293 lines for eukaryotic recombination tests were: a previously generated HEK293-Cre stable transfected line (neomycin resistant – Neo^r^) and a newly generated HEK293-Dre (hygromycin resistant – Hygro^r^) line, built by Flp-mediated insertion of a pcDNA5/FRT-Dre expression vector into the HEK293-FlpIn cell line, using manufacturer recommended procedures (Flp-In system, Invitrogen). In this strategy, the Dre cDNA is inserted as a single copy into the HEK293 genome, downstream of a CMV promoter, together with the Hygro^r^ cassette. Several colonies were selected under hygromycin, and tested for Dre recombination with the WT proxP-R-roxP-G vector described above. Clone 4 was used in the studies described below. The eukaryotic expression vectors containing combinations of mutant and WT roxP sites were transfected onto duplicate poly-D-lysine (Sigma-Aldrich, P6407, 0.5 µg/µl in water) coated coverslips of either HEK293-Dre (clone 4) or HEK293-Cre cells, using a branched polyethylenimine transfection reagent (Sigma-Aldrich, cat # 408727, 1 µg/µl) (generated and optimized by Jung-Woong Kim, N-NRL/NEI). After 48 hr, cells were fixed for 10 min in 4% paraformaldehyde, counterstained with DAPI, and mounted in Fluoromount-G (Southern Biotech) onto slides. Cells were imaged with a 20 × objective using a Zeiss Axioimager.Z2 fitted with an Apotome, and a MRM Axiocam. For each construct and HEK293 line, six 0.15 mm^2^ (full camera field) images were taken using the same set of fluorescence intensities and exposure time settings across the entire experiment. All images were collected with identical settings for fluorescence intensity and exposure times. However, red fluorescence signals in Figure 6 B columns 1, 2, and 5, were much dimmer then in all other conditions, so the maximum value of the red lookup table was adjusted to one-fifth of its initial value for these panels. Images were imported and analyzed in ImageJ, using the LOCI bioformats plugin (Open Microscopy Environment, http://www.openmicroscopy.org/site/products/bio-formats), and red, green, and yellow (double positive) cells were counted using the cell counter plugin (Kurt De Vos, University of Sheffield, Academic Neurology, kurt.devos@iop.kcl.ac.uk) over six individual 20 × fields derived from two distinct transfected coverslips for each of the experimental conditions in Figure 6, B and C, and Figure 7, C and D. For prox-R-rox G experiments, green and yellow (double positive) cells were considered as recombined and normalized to the total number of transfected cells (sum of red, green, and yellow cells).

For pAAVFREX experiments, red and yellow (double positive) cells were considered as recombined and normalized to the total number of transfected cells (sum of red, green, and yellow cells). For the pAAVPTPY construct, TFP and YFP positive cells were considered recombined and normalized to the number of DAPI cells in the field (pAAVPTPY does not express any reporter in the absence of recombination).

### Statistical analysis

Comparison of nucleotide frequency distribution in the p2NKanP vector before and after Dre recombination (see legend for Figure 2) was performed using χ^2^ statistics with a custom written Matlab (Mathworks, Inc.) procedure, as follows. Actual A, G, C, and T frequencies for each of the four mutated positions (2, 3, 6, and 7) were tabulated for the 82 Dre-treated minipreps and 24 untreated controls. Expected frequencies for each position (sample), treatment and nucleotide (category) were calculated, and then the χ^2^ statistics and significance value for each position were derived, using built-in Matlab functions, given three degrees of freedom [(two treatments−1) × (four categories−1)]. The null hypothesis was defined as the two sets of frequencies for each position being derived from the same distribution. The χ^2^ tables are reported in Supporting Information, Table S1, and the Matlab script is provided in File S1.

Box-whisker plots and statistical analyses were performed in Matlab. Table S2 shows medians, means, number of replicates, images taken, and cells counted in each experiment, as well as outcomes of Student T tests and Kolmogorov–Smirnov tests for comparisons drawn in Figure 7. Explanation of Box Whisker plots: the tops and bottoms of each ‘‘Box’’ are the 25^th^ and 75^th^ percentiles of the samples, respectively. The distances between the tops and bottoms are the interquartile ranges. The line in the middle of each box is the sample median. Whiskers are drawn from the ends of the interquartile ranges to the furthest observations within the whisker length (the adjacent values).

### Data availability

All reagents generated and described in this work are available upon request from the corresponding author.

## Results

### Library and vector design for Dre recombination assay

We used the existing information on the loxP site to guide our analysis of the roxP spacer region. Since the equivalence of the spacer positions on the two target sites was not immediately obvious, we designed two random nucleotide libraries, carrying random base pairs in the roxP spacer and surrounding bases (Figure 1A [top] and Figure 1B). The rox4R library (spacer positions 3–6) would contain 4^4^ = 256 possible combinations. The rox6R library (positions 2–7) covers the roxP spacer and the first adjacent base of the inverted repeat on either side, which would allow us to test the prediction that positions 2 and 7 are the functional equivalents of those in the loxP spacer, and would contain 4^6^ = 4096 variants. Given that this number of combinations would be difficult to cover in a screen for the desired mutants, we also generated a library that covers two bases at the junction between each inverted repeat and the spacer (rox2N library, positions 2, 3, 6, and 7, Figure 1A [top], and Figure 1B), but expected to have a smaller search space (256 variants). The three libraries, and a WT positive control roxP were inserted at the roxA site of the proxA-Kan^r^-roxB recombination target vectors (Figure 1, C and D). Upon successful Dre-mediated recombination, the kanamycin resistance gene is eliminated, resulting in a vector containing just the ampicillin resistance cassette and the origin of replication (Figure 1C, bottom right). The circular recombination product including the Kan^r^ is eliminated in subsequent bacterial cell divisions since it does not have a replication origin.

### Recombination test strategy

The p4RKanP, p6RKanP, and p2NKanP libraries, and the pPKanP WT control, were assayed for Dre recombination by cotransformation with pGB2-Dre (Spc^r^) into DH5α, and plated on spectinomycin^+^ ampicillin^+^ plates, ensuring selection for both Dre and target vectors, without selecting against recombination (Figure 2A). Since the pMB1 origin of the target vectors replicates at more than 75 copies per bacteria, we reasoned that unrecombined targets might be mixed with recombined ones in the same colony, and therefore did not test individual colonies from this step. Rather, we pooled all colonies from the plates (around 2000 colonies for p4RKanP and p2NKanP, and 6000 colonies for p6RKanP), allowed them to amplify, extracted DNA from the common mix, and digested an aliquot of this pooled DNA with *Pvu*I + *Pst*I (Figure 2, B and C). Whereas the pPKanP WT roxP control and the p4RkanP library showed a nearly complete recombination pattern (∼0.9), the p6RKanP and p2NkanP libraries showed significantly less recombination (∼0.3 and 0.1, respectively, quantitated in Figure 2C). These results suggest that the two positions flanking the spacer (positions 2 and 7, Figure 1A) have a much stronger influence on the ability of the mutant to recombine with the WT roxP, while substitutions in the central 4bp of the roxP site affect compatibility with the WT roxP in a less significant manner. Thus, it appears that the desired mutants might be present in the p6RKanP and p2NKanP libraries, and that the crossover region of the roxP site might extend beyond the 4 bp central asymmetric motif (positions 3–6).

In order to select mutant spacer sequences incompatible with the WT, the p2NKanP library was cotransformed with pGB2-Dre (Figure 2A) and selected on spectinomycin^+^ kanamycin^+^ plates. Purified DNA from the pooled colonies was retransformed and individual colonies were amplified under kanamycin selection. Colonies were analyzed by restriction digestion, sequenced, and compared to sequences from the input, unrecombined p2NKanP library (Figure 2D). The nucleotide distributions at spacer positions 2 and 7 were significantly skewed in the spacers derived from the screen when compared to the WT library, while distributions at positions 3 and 6 were relatively unaffected by the selection process (Figure 2D and Table S1; statistical significance assessed for each spacer position by χ^2^ test, see *Materials and Methods*). Position 2 had a strong bias toward G (41/82 = 50% after, compared to 8/24 = 33.3% before recombination) and C (33/82 = 40.24% after, compared to 5/24 = 20.83% before recombination). Position 7 had a strong bias toward G (40/82 = 48.8% after, compared to 5/24 = 20.83% before recombination). Thus a mutation consensus could be derived for the roxP spacer as 1–8 = Ag/cGCCggT, keeping in mind that, in this screen, positions 1 (A), 4 (C), 5 (C), and 8 (T) were kept WT. Of the four mutated positions, the WT nucleotide was selected against only at position 2 (T, 7/82 = 8.53% after, compared to 4/24 = 16.67% before recombination). Since we were interested in the spacers that are least likely to interact with the WT, we identified the variant spacers that occurred more than once (tabulated in Figure 2E). Five mutant spacer sequences occurred three times in our screen, while only one spacer sequence occurred four times (AgGCCtgT). Thirteen spacer sequences occurred twice, among them the mutant consensus, AgGCCggT. This indicates a level of enrichment for certain spacers, as the expected probability of any given spacer occurring four or three times, respectively, in 82 draws would be (82/256)^4 = 0.01, and (82/256)^3 = 0.03. Among the seven spacers selected for further analysis (Figure 2E), only rox7 (AgGCCAgT) was also found (once) in the unrecombined control sequences. Thus, although the sequencing depth for both the control and selected libraries were not sufficient to cover the expected diversity, it allowed us to identify a reasonable number of candidates for roxP incompatible sites, and confirmed our prediction that mutations at positions 2 and 7 are deleterious for roxP compatibility.

### Incompatible mutant spacer validation

We picked representative clones for each of the enriched mutant spacers, as well as the mutation consensus (Figure 2E, last row), and retested them in our recombination assay. Henceforth, the mutant spacer sequences will be identified by the representative clone number, *e.g.*, AgGCCtgT, present in clone number 12 is rox12 (Figure 2E). The plasmids carrying the seven rox mutant spacers rox7–rox85 upstream and a WT roxP site downstream of the Kan^r^ cassette (p7KanP – p85KanP, Figure 3A) were subjected to the Dre recombination assay (Figure 3). The diagnostic restriction digest revealed a nearly complete lack of recombination for all tested mutant rox spacers, while the positive control pPKanP vector showed full recombination (Figure 3, B and C), despite the persistence of the Dre expression vector in the bacterial isolate (5.3 kb band, Figure 3B). In addition, purified DNA from the two independent colonies was transformed on ampicillin^+^ plates, and then replica plated onto kanamycin^+^ plates (Figure 3D and Figure S2). As expected, no Kan^r^ colonies were observed for the control pPKanP plasmid, while most Amp^r^ colonies from the mutant spacer constructs (p7KanP–p85KanP) were successfully replicated onto the kanamycin^+^ plates (Figure 3D, Figure S2, and quantitation in last column of Figure 3A).

### Testing self-recombination efficiency of identified roxP spacers

To test the ability of the identified mutant spacers to engage in Dre-mediated recombination, we generated constructs in which the roxA and roxB sites, encompassing the Kan^r^ gene, were occupied by the same rox mutant spacer. We conducted this test for spacers rox7, rox8, rox12, rox61, and rox85, which had the highest degree of identity with our derived consensus, and compared them to the roxP WT control (Figure 4A). After cotransformation with pGB2-Dre and selection on spectinomycin^+^ ampicillin^+^, two individual colonies were picked and analyzed as described in the previous section (Figure 4, B and C, and Figure S3A). The complete loss of the unrecombined (1.8 kb) band in the diagnostic restriction digest, and essentially complete loss of kanamycin resistance in replica plating tests, indicated that the five chosen mutant spacers are able to recombine with themselves with efficiencies comparable to the WT control in the prokaryotic environment. Together with results presented in the previous section, this suggests that the lack of recombination with the WT roxP is a result of spacer incompatibility and not of lack of intrinsic ability to participate in the recombination reaction.

### Testing cross-talk between novel rox variants and Cre recombinase

To address the possibility of cross-talk between the Cre recombinase and the newly generated mutant sites, we transformed our five rox mutant plasmids and the WT control in a bacterial strain expressing the Cre recombinase (BS1365), which we and others have routinely used to test Cre-loxP recombination in target vectors (*e.g.*, Siegel *et al.* 2001, and data not shown). As seen in Figure 4, D and E, and Figure S3B, no visible recombination of either mutant or WT rox target sites was detected by either diagnostic digest or replica plating. This confirms the many previous reports that there is little reactivity between the Dre and Cre recombination systems, and that our newly identified spacers did not acquire Cre sensitivity by virtue of the substituted bases.

### Testing recombination efficiencies of mutant rox sites in a purified system

Bacterial recombination assays were powerful for the screening and evaluation of identified mutants. However, saturation effects could overestimate recombination efficiencies seen for the identified mutant spacers. We therefore used a purified system to directly compare self-recombination efficiencies of the identified mutants and WT roxP sequence. The same set of vectors used in the prokaryotic self-recombination test (Figure 4A) were subjected to Dre recombination using bacterially expressed and affinity purified Dre protein, and reaction conditions similar to those used for equivalent *in vitro* assays for the Cre-loxP purified system (Lee and Saito 1998; Ghosh and Van Duyne 2002). The vectors were linearized, and then subjected to recombination for 5, 10, 15, 20, 30, 60, and 120 min, with around 10-fold molar excess Dre. For diagnostic and quantitative purposes, the DNA was further digested with *Pvu*I and *Msc*I, loaded on agarose gels and quantitated by gel densitometry. The characteristic recombination bands (see Figure 5, legend) were normalized to the recombination-indifferent internal control band, and reported in percent.

We find that *in vitro* self-recombination efficiencies for the five analyzed mutants vary significantly, with spacer rox8 exhibiting about 0.7 and spacers rox7, rox12, and rox61 about 0.4–0.5 of WT recombination efficiency (Figure 5, B–D).

### Testing mutant rox sites in eukaryotic cells

We next established an assay to test the recombination specificity and efficiency of our rox mutants in eukaryotic cells. A Dre-expressing HEK-293 stable cell line was established using a single copy integration system based on Flp recombination (see *Materials and Methods*). In addition, a roxA-mCherry-STOP-roxB-eGFP cassette was inserted downstream of a CMV promoter in the pcDNA3.1 eukaryotic expression vector (Figure 6A, prox-R-rox-G). Using this strategy, we tested two mutants, rox12 (most frequently recovered spacer) and rox85 (consensus noncompatible sequence), for the ability to self-recombine and the lack of recombination with the WT locus. The vectors (Figure 6A, right table) were individually transfected in either the HEK293-Dre cell line (Figure 6, B, D, and F), or a previously generated HEK293-Cre line (Figure 6, C and E), and analyzed by direct fluorescence 48 hr after transfection. We found that rox12 and rox85 mutant rox sites were capable of self-recombination at efficiencies comparable to the WT roxP (Figure 6, B, D, and F, columns 1, 2, and 4). HEK293-Dre cells transfected with the proxP-R-roxP-G, prox12-R-rox12-G, and prox85-R-rox85-G vectors did not show a complete conversion from red to green fluorescence, with more than half the cells exhibiting both. However, whereas images were taken with identical fluorescence intensity and exposure time settings across all experiments, the red fluorescence intensity was much lower for images in Figure 6B, columns 1, 2, and 4, compared to the rest of the images and had to be digitally enhanced for counting or visualizing transfected cells. This suggests that mCherry levels were very low in HEK293-Dre cells transfected with the three vectors capable of self-recombination, perhaps as a result of residual expression of mCherry from target plasmid before recombination. Nevertheless, 5% of prox12-R-rox12-G and 8.7% of prox85-R-rox85-G transfectants were GFP negative (Figure 6B, quantitated in Figure 6, D and F) while less than 1% of proxP-R-roxP-G transfected HEK293-Dre cells had not undergone any detectable recombination. Thus, recombination mediated by these two newly identified rox spacers is slightly less efficient than that for the WT. However, neither rox12 nor rox85 showed any detectable recombination with the WT roxP (Figure 6, B, D, and F, columns 3 and 5). In addition, none of the WT–mutant or mutant–mutant combinations resulted in detectable recombination when transfected into the HEK293-Cre line (Figure 5, C and E). In contrast, a control FLEX cassette type vector (using loxp and lox2272 sites) expressing membrane-bound eYFP and PSD95-TFP in a Cre-dependent manner (B. Wu and T. C. Badea, unpublished results) showed fluorescently labeled cells when transfected in HEK293-Cre but not HEK293-Dre-expressing cells (data not shown and Figure 7, C–G). Thus, the two novel rox sites identified in the bacterial recombination screen exhibit specific, efficient self-recombination when exposed to the Dre recombinase, and no cross-reactivity with the Cre recombinase when tested in eukaryotic cell culture.

Since the rox12 self-recombination efficiency more closely matched the WT control, we used the rox12 and roxP sites to generate a sequential inversion–excision Dre recombination cassette, analogous to the FLEX cassettes used for the loxP system. cDNAs for mCherry and eGFP arranged in reversed configuration and separated by a bidirectional SV40 polyA transcription STOP signal were placed between two sets of rox12-roxP sites, arranged in inverted configuration with respect to each other (Figure 7A). This cassette is designed to express eGFP before and mCherry after Dre recombination. Two alternative, reversible inversions, followed by irreversible excisions, result in an irreversible inversion of the fluorescent protein cassette and mCherry expression (Figure 7B). When tested in our HEK293-Dre line, the FREX cassette showed more than 95% recombination efficiencies, at levels similar to the WT roxP-mCherry-STOP-roxP-eGFP configuration (Figure 7, C, E, and G). However, no recombination was detected in HEK293-Cre cells (Figure 7, D, F, and G). In contrast, the Cre-dependent FLEX vector, pAAVPTPY, resulted in recombination in HEK293-Cre but not HEK293-Dre cells (Figure 7, C–G).

## Discussion

We report here the identification of several roxP variants that are incompatible with the WT Dre recombinase roxP target site, but recombine efficiently with themselves. We used a random nucleotide insertion strategy to map the involvement of base pair positions in and around the roxP asymmetric spacer in Dre-mediated recombination specificity. We find that, whereas mutations covering the central 4 bp have a mild effect on WT compatibility, mutations involving the bases adjacent to the roxP spacer, specifically in the first base of the inverted repeat, drastically reduce rox mutant-roxP WT recombination. Most of the identified mutants show three or more base substitutions compared to the WT roxP consensus. WT incompatible sites tend to have strong preferences for G/C and G, respectively, in the bases flanking the unidirectional spacer.

### Implications for the Dre-roxP mechanism

Despite the good conservation between Dre and Cre proteins and between roxP and loxP sites (Guo *et al.* 1997; Sauer and McDermott 2004), the task of mapping the previously identified base pair functions of the loxP site onto the roxP site is more difficult than expected. The major challenge consists in the smaller asymmetric spacer of the roxP site (4 bp *vs.* 8 bp in loxP). The loxP spacer’s compatibility with the WT is significantly affected by substitutions at the G-C base pair 5′ to the catalytic attack site (Figure 1A, position 7), less perturbed by substitutions at positions 3–6, and essentially unaffected by mutations at positions 1 and 8 (Lee and Saito 1998). The backbone phosphate bonds between nucleotides 7 and 8 on the bottom strand, and subsequently nucleotides 1 and 2 on the top strand, are the catalytic attack sites that form transient covalent bonds with tyrosine Tyr324 of Cre and electrostatic bonds with the other four bases of the catalytic pocket (Arg173, His289, Arg292, and Trp315) (Guo *et al.* 1997). If the bases responsible for spacer compatibility are recognized by virtue of their asymmetry, one might infer that the equivalent positions for the Dre-roxP system lie within the four central base pairs of the roxP site (positions 3–6 in Figure 1A). However, if the effects on spacer compatibility are dictated by their positions relative to the cleavage site, and can be extrapolated to the Dre-roxP complex, our data argue that the roxP crossover region, and hence the cleavage site, reaches at least into the first base of the inverted repeat, since mutations including spacer bases 2,3 and 6,7 (Figure 1A) had dramatically more impact than mutations restricted to positions 3 through 6. This may suggest that the roxP crossover region is actually closer in length to loxP. These predictions are consistent with our atomic structure model of the Dre-roxP interaction based on a previously published crystal structure of the Cre-loxP complex (Guo *et al.* 1997) (Figure S1) using homology modeling. These findings lend support to the hypothesis that functionally the roxP and loxP crossover regions are similar in length, and that strand cleavage happens at equivalent positions (1–2 top and 7–8 bottom) in both (Sauer and McDermott 2004). Since the mapping of the roxP spacer was only accessory to our main goal, we have not further dissected these questions. Clearly, more mutational analysis and other complementary approaches, like the use of suicide substrates and crystalography, are needed to definitively identify the Dre catalytic target onto the roxP spacer. However, our analysis offers a useful starting point.

### Specificity and recombination efficiency

Out of the identified roxP incompatible variant spacers, the seven most frequently encountered sequences showed essentially no cross-reactivity with the WT roxP site upon retesting. Six variants had guanine nucleotides at positions 2 and 7 in the spacer (Figure 2D), while a seventh had cytosine nucleotides on either side (rox8), in keeping with the noncompatible consensus derived from our screen (Ag/cGCCggT, Figure 2D). In fact, similar observations have been reported in screens for loxP-incompatible lox sites (Lee and Saito 1998; Siegel *et al.* 2001; Langer *et al.* 2002; Missirlis *et al.* 2006; Jung *et al.* 2007), in which self-compatible, efficiently recombining sequences tend to prefer G or C at positions 2 and 7 (Langer *et al.* 2002; Missirlis *et al.* 2006), and the general recombination efficiency is associated with the presence of G bases (Missirlis *et al.* 2006). However, it should be pointed out that in the saturation screen performed in Missirlis *et al.* (2006), the spacers exhibiting the highest frequency of self-recombination also had a high degree of promiscuity Figure 5 in (Missirlis *et al.* 2006), but that most of that promiscuity may be with other G containing spacers, since sequences containing Gs at positions 2 and 7 were less likely to recombine with spacers containing other nucleotides at those positions, Figure 6 in (Missirlis *et al.* 2006). In this context, it would be interesting to explore the mutual recombination compatibility of our novel rox sites. If differences at positions 2 and 7 are a predictor of spacer compatibility, six of the further characterized spacers that carry Gs at both positions are very likely to cross-react. Since mutant rox8 (AcGCCtcT) carries Cs in positons 2 and 7, it is the most likely to be incompatible with the other newly identified rox variants. The five rox variants tested for self-recognition in the bacterial system (rox7, rox8, rox12, rox61, and rox85) showed nearly complete recombination patterns. However, the time course analysis performed with purified components reveals that all identified spacers have reduced self-recombination efficiency when compared to the WT, with rox8 exhibiting the highest self-recombination efficiency among the novel mutant sites (∼70%), while three others (rox7, rox12, and rox65) are around 40%, consistent with the findings in the Cre-loxP system, in which substitutions at positions 2 and 7 had reduced self-recombination properties (Lee and Saito 1998). For the two spacers assayed in eukaryotic tissue culture, rox12 (the most frequently recovered rox variant from the screen) and rox85 (representing the consensus site), recombination specificity was high (no cross-talk with the WT) but the efficiency of self-recombination was somewhat reduced compared to the WT control, as judged by both the number of remaining unrecombined cells and the degree of complete conversion from the unrecombined to recombined forms.

The assay-dependent differences in self-recombination efficiency may be a measure of relative amounts of Dre protein to roxP targets available and the distinct time courses employed in the purified, bacterial and eukaryotic systems. Whereas the purified component, biochemistry experiment seems to get close to the maximal levels at around 30 min for roxP and most of its variants, the bacterial recombination assay has 24–48 hr and the eukaryotic assay 48 hr of potential interaction between less well understood amounts of Dre and target sites. Although the *in vitro* 10-fold molar excess sounds like a large margin, in the bacterial experiment, the number of targets/bacterium should be equivalent to the maximum number of plasmid copies, (∼75 for pMB1), and we have not assayed the amount of Dre generated from the pSC101 plasmid (1–5 plasmid copies/bacteria, and lac promoter). Moreover, the small circular product, devoid of replication origin, will be gradually diluted during cell divisions, favoring the excision reaction and complete recombination. However, recombination assays using roxP WT target vectors but coexpressing Dre from a high copy number vector (300–500 copies/bacteria) resulted in abnormally recombined products (data not shown), suggesting that the amount of Dre protein delivered can be critical not only to the efficiency but also to the specificity of the reaction. In the eukaryotic system, the HEK293-Dre line expresses Dre from a single copy knock-in locus using a CAG promoter, and hence protein levels are expected to be smaller than those for transient transfection or infection conditions. In addition, the fluorescent protein reflecting the unrecombined state (*e.g.*, mCherry) could be expressed and accumulated in transfected cells before recombination and conversion to eGFP. However, it is clear that the identified spacer mutations have an impact on the recombination efficiency, consistent with findings for lox2272 and lox511 (lox51), the most widely used loxP substitutes (Lee and Saito 1998; Siegel *et al.* 2001). Although rox8 was the most efficient variant in the *in vitro* self-recombination assay, it also exhibited a small amount of roxP WT cross-talk in the cotransformation assay, and hence we opted for rox12 (and the least efficient rox85 as a control) as our further candidate in eukaryotic tests.

### Is there cross-talk between the Dre and Cre systems?

We do not find any evidence for cross-talk between Cre recombinase and the newly identified roxP target sites or the WT control. This is in agreement with observations from many labs that have reported no cross-activation between the Dre and Cre systems in *E. coli*, HEK293 cells, zebrafish, mouse embryonic stem cells, or mouse lines carrying either Dre- or Cre-expressing transgenes and suitable reporters (Sauer and Henderson 1988; Anastassiadis *et al.* 2009; Park and Leach 2013; Sajgo *et al.* 2014). However, recently two studies have reported cross-activation between Dre and Cre systems, especially when the Dre or Cre recombinase and/or the reporter constructs are delivered to HEK293 cells or hippocampal or cortical neurons via AAV vectors (Fenno *et al.* 2014; Madisen *et al.* 2015). In fact, Madisen *et al.* found little cross-talk between the two systems when the same genetically encoded reporter was tested by crossing with single copy targeted Dre and Cre drivers (compare Madisen *et al.* 2015, Supplementary Figure 3B to Supplementary Figure 4B). In addition, this could be due in part to the choice of mutations in the target roxP sites, Figure 1 in (Fenno *et al.* 2014), which bring up the homology between Dre and Cre left hemisites to 13/16 nucleotides. In our hands, nonspecific recombination, even in the absence of cognate target sites, can be achieved when large amounts of recombinase are expressed (see above paragraph). We therefore think that the relative amounts of recombinases and target vectors expressed could be the reason for the nonspecific effects seen in the viral delivery experiments, but not in full embryo genetic manipulations.

### Potential applications in genetic manipulations

The use of SSRs in molecular genetic manipulations is rendered tremendously powerful by its ability to generate combinatorial expression patterns restricting gene manipulations to increasingly narrower domains (Jensen and Dymecki 2014). Dre could function as one of the members of the combinatorial recombination code. In many instances, it is desirable to use two distinct recombination target sites in order to be able to intersect three gene expression domains and single out one cell population. This type of manipulation has been implemented at genetic loci for the Dre, Flp and Cre recombinases in mice and zebrafish (Farago *et al.* 2006; Hirsch *et al.* 2013; Park and Leach 2013; Sajgo *et al.* 2014; Madisen *et al.* 2015), and for several new recombinases in AAV-based strategies (Nern *et al.* 2011; Suzuki and Nakayama 2011; Karimova *et al.* 2013; Fenno *et al.* 2014). The strategy may be arranged “in parallel”, by having the two recombinases expressed from two different genetic elements, with a third locus expressing a dual reporter, which sequentially switches color in response to either one or both recombinases (Farago *et al.* 2006; Hirsch *et al.* 2013; Park and Leach 2013; Madisen *et al.* 2015). It could also be arranged in a “serial” fashion, involving a first genetic locus driving one of the recombinases which then induces the expression of the second recombinase from a distinct locus, followed by the final activation of a reporter dependent on a third gene (Sajgo *et al.* 2014).

Constructs in which expression of a reporter is activated by a recombinase have been arranged in variants of two basic designs. The “Promoter – loxP – STOP – loxP – Reporter” design (Lobe *et al.* 1999; Badea *et al.* 2009), as seen for instance in the vectors in Figure 6, will prevent expression of the reporter by transcriptional termination at the STOP signal until Cre recombination occurs. Although successfully used in many occasions, it is often flawed by incomplete transcription termination at the STOP, resulting in some reporter being made even in the absence of Cre (Badea *et al.* 2009; Sajgo *et al.* 2014). This inconvenience can be overcome by the FLEX design, in which two incompatible lox sites (lox2272 and loxP) are arranged in tandem and in reverse orientation on either side of an inverted (inv) reporter [Promoter – lox2272 – loxP – inv(Reporter) – inv(lox2272) – inv(loxP)] (Schnutgen *et al.* 2003; Atasoy *et al.* 2008). Cre induces two alternative inversions followed by excisions resulting in the same final configuration: Promoter – lox2272 – Reporter – inv(loxP). This strategy, first used in a mouse model, and extremely popular for Cre-dependent AAV reporters, relies heavily on the incompatibility between the WT loxP site and the mutant lox2272 site. Our novel rox sites can be used for the same type of application in combination with roxP for the Dre recombinase. We propose the analogous FREX cassette for the Dre to roxP system as demonstrated for the roxP-rox12 combination, or extended to the rox8 or other mutants identified in the screen. Since these mutants had zero recombination with the WT and were not targeted by the Cre recombinase, they may represent an improvement over a recently published alternative rox site (Fenno *et al.* 2014), and would be ideal in future combinatorial genetic manipulations involving Dre. Regardless of the specific configuration of the adopted strategy and/or the specificity and efficiency of the employed recombinase and specific target sites, our results add to the many previously published biochemical data that highlight the importance of the level of expression of the recombinase and the abundance of the target sites in the outcome. These aspects are particularly critical in genetic applications, where driver loci have intrinsic cell type specificities, time courses and levels of expression, while target loci have distinct tolerance for recombination, all properties which are somewhat predetermined and have to be carefully matched in order to achieve the desired level and specificity of recombination.

## Supplementary Material

Supporting Information
