# Supplementary material for: Novel Heterotypic Rox Sites for Combinatorial Dre Recombination Strategies
Source: G3 (Bethesda). 2015 Dec 29;6(3):559–71. doi: 10.1534/g3.115.025841 (PMC4777119; doi:10.1534/g3.115.025841)
Supplement: Supporting Information [file supp_6_3_559__index.html]

Novel Heterotypic Rox Sites for Combinatorial Dre Recombination Strategies — Novel Heterotypic Rox Sites for Combinatorial Dre Recombination Strategies — Supporting Information 

# Novel Heterotypic Rox Sites for Combinatorial Dre Recombination Strategies

## Supporting Information for Chuang *et al.*, 2016

**Files in this Data Supplement:**

- Figure S1 - Model of the Dre-roxP complex based on the Cre-loxP crystal structure. (.pdf, 455 KB)
- Figure S2 - Confirmation of isolated roxP incompatible mutants. (.pdf, 413 KB)
- Figure S3 - Novel roxP sites show efficient self recombination in response to Dre but not Cre recombination. (.pdf, 563 KB)
- Table S1 - Chil Square calcualtions for experiment in Figure 2D. (.pdf, 61 KB)
- Table S2 - a, b, Medians, Means, Standard deviations, number of images, individual cells quantified, and independent transfections for experiments described in Figure 2 (a) and Figure 3 (b). (.pdf, 591 KB)
- File S1 - Supplementary methods. (.pdf, 86 KB)
