# Supplementary material for: Novel Heterotypic Rox Sites for Combinatorial Dre Recombination Strategies
Source: G3 (Bethesda). 2015 Dec 29;6(3):559–71. doi: 10.1534/g3.115.025841 (PMC4777119; doi:10.1534/g3.115.025841)
Supplement: Supporting Information [file supp_g3.115.025841_FigureS1.pdf]

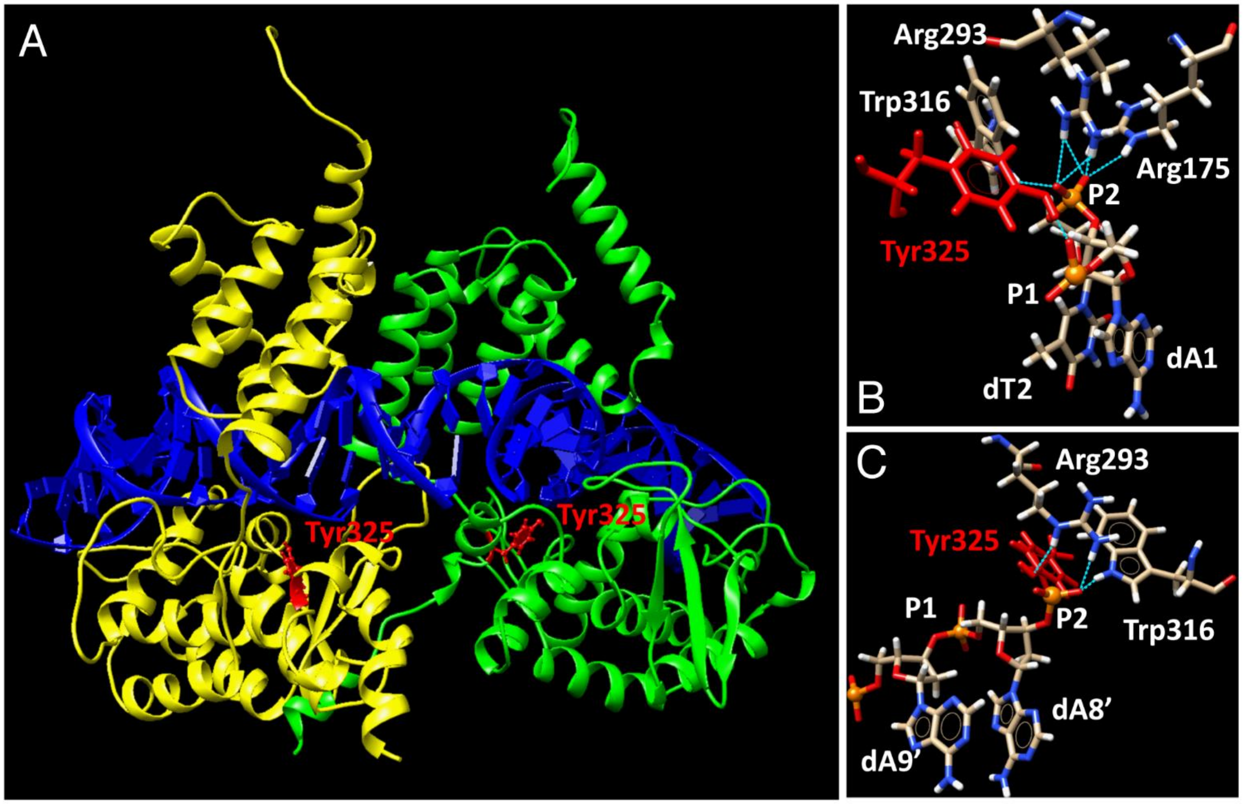

Figure S1: Model of the Dre-roxP complex based on the Cre-loxP crystal structure.

The model is based on Dre and roxP sequences superimposed on the solved crystal structure of a Cre-loxP catalytic intermediate (GUO *et al.* 1997), in which one Cre subunit has cut the bottom strand of the loxP site between bases Guanine 8 (G8') and Cytosine 7' (Figure 1A), and forms a covalent 3'-phosphotyrosine intermediate between Tyrosine 324 and G8'. The second Cre subunit is noncovalently bound to the second inverted repeat of the loxP site (reference (GUO *et al.* 1997) Figures 2 and 4).

A, Ribbon model of two Dre proteins bound to one roxP site. The Dre subunit corresponding to the covalent 3'-phosphotyrosine intermediate is in yellow, while the noncovalently bound Dre

subunit is in green. Side chains of active site Tyr325 are in red and roxP DNA in blue. B, C, Enlarged views of the Dre-roxP active sites modeled on the Cre subunits that have (C) or have not (B) cleaved the DNA. Of the five predicted active site aminoacids, Arginine 175, Arginine 293, Tryptophan 316, and Tyrosine 325 show distances consistent with interactions with the oxygen atoms of the P2 and P1 phosphate groups (GUO *et al.* 1997; SAUER and MCDERMOTT 2004), and are shown in B and C. His290, which was not close enough to allow any possible bonds, was omitted. Arg175 was omitted from panel C for clarity although it has predicted interactions with P2. Nucleotides are numbered according to spacer positions in Figure 1A, and followed by apostrophes for the bottom strand. P1 is the 5' Phosphate of dA1 (B) or dA8' (C). The phosphate group P2 connects nucleotides Adenine 1 (dA1) and Thymidine 2 (dT2) of the non-cleaved roxP half-site (B), and is predicted to connect Tyr325 to the 3' Hydroxyl group of Adenine 8' (dA8') on the bottom strand of the cleaved roxP half-site (C). Note that in the original Cre-loxP structure (Pdb file: 1Crx) the phosphate P2 of the cleaved loxP strand (C) is covalently bound to both Tyr324 (Cre) and the rybose of G8'. Coordinates of P2 atoms from the Cre structure were transformed and transferred to our Dre-roxP complex to model a possible pseudo-covalent bond between the dA8’ ribose and the P2 phosphor atom (C). Light blue stippled lines represent predicted hydrogen bonds.
