# Supplementary material for: Novel Heterotypic Rox Sites for Combinatorial Dre Recombination Strategies
Source: G3 (Bethesda). 2015 Dec 29;6(3):559–71. doi: 10.1534/g3.115.025841 (PMC4777119; doi:10.1534/g3.115.025841)
Supplement: Supporting Information [file supp_g3.115.025841_FigureS2.pdf]

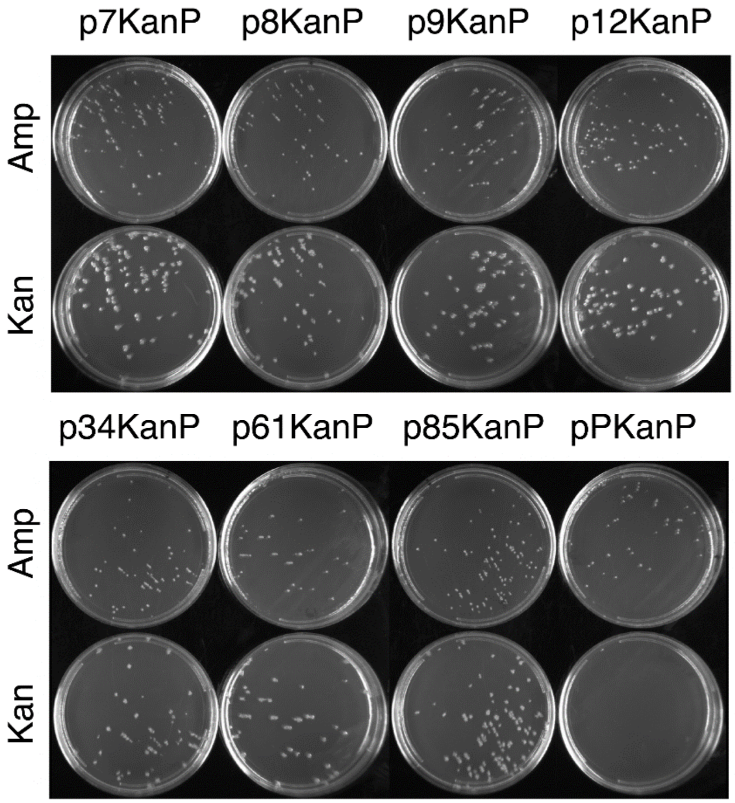

Figure S2: Confirmation of isolated roxP incompatible mutants.

Ampicillin plates and replicas onto kanamycin plates are the full versions of the insets described in Figure 3D. All seven mutant spacers (p7KanP through p85kanP) show conserved kanamycin resistance, and hence lack of recombination with the wild type roxP site, while full recombination resulting in loss of kanamycin resistance is seen for the wild type control.
