# Supplementary material for: Novel Heterotypic Rox Sites for Combinatorial Dre Recombination Strategies
Source: G3 (Bethesda). 2015 Dec 29;6(3):559–71. doi: 10.1534/g3.115.025841 (PMC4777119; doi:10.1534/g3.115.025841)
Supplement: Supporting Information [file supp_g3.115.025841_FigureS3.pdf]

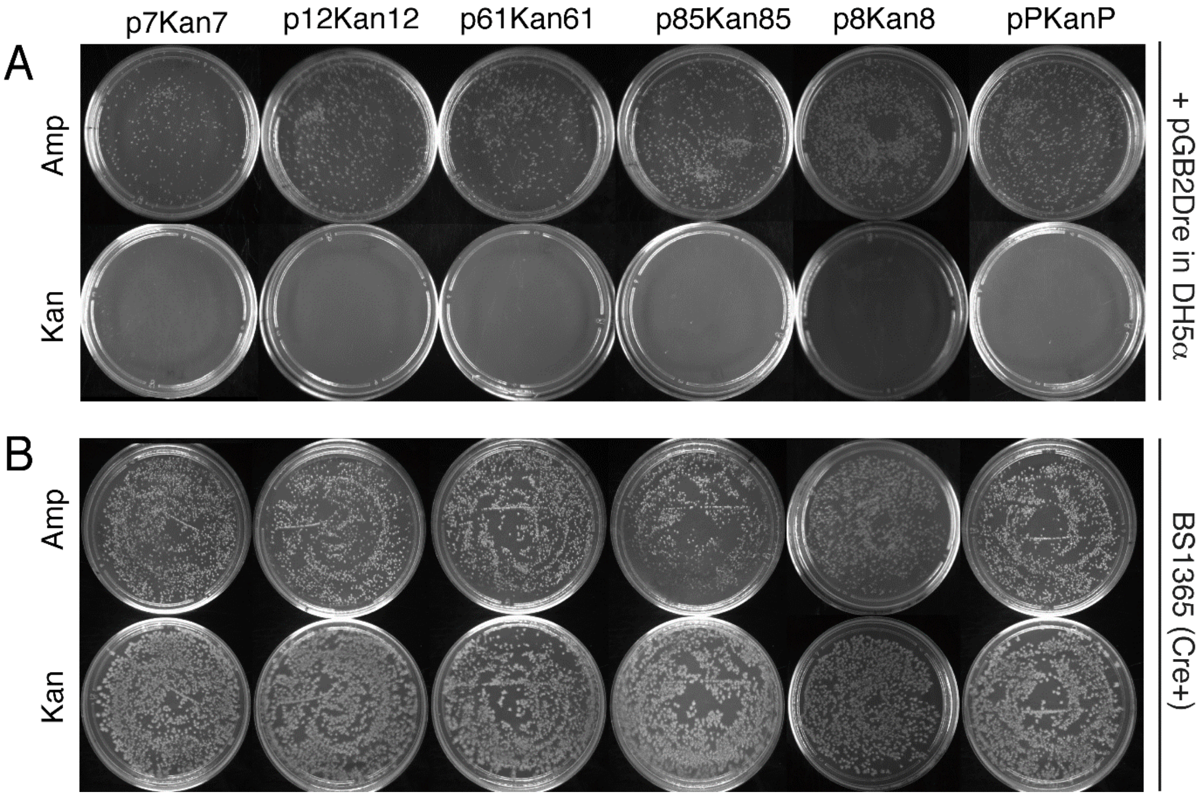

Figure S3: Novel roxP sites show efficient self recombination in response to Dre but not Cre recombination.

A, Replica plating for experiments described in Figure 4 B and C. Note that essentially no surviving colonies are seen after replica plating of the ampicillin resistance colonies onto kanamycin plates, indicating that essentially all novel heterotopic sites supported self-recombination and therefore loss of the kanamycin resistance marker as a result of exposure to Dre.

B, Replica plating experiments of the same heterotopic targets and wild type control after transformation in Cre expressing bacteria (BS1365) demonstrates equal survival on ampicillin and kanamycin, and hence resistance to recombination by the Cre recombinase (see Figure 4, D,E).
