# Supplementary material for: Novel Heterotypic Rox Sites for Combinatorial Dre Recombination Strategies
Source: G3 (Bethesda). 2015 Dec 29;6(3):559–71. doi: 10.1534/g3.115.025841 (PMC4777119; doi:10.1534/g3.115.025841)
Supplement: Supporting Information [file supp_g3.115.025841_TableS1.pdf]

Table S1.
Chil Square calcualtions for experiment in Figure 2D
Degrees of freedom = (columns l 1) * (rowsl 1)
Null hypothesis l data before and after Dre are derived from the same distribution

Chi Square table - Position 2

position2 actual	before	plus Dre
A	4	1
T	7	7
G	8	41
C	5	33
position2 expected	before	plus Dre
A	1.13208	3.867925
T	3.16981	10.83019
G	11.0943	37.90566
C	8.60377	29.39623
	twosided	
P value	0.00036	
ChiStat	18.4415	
Degrees	3	

Chi Square table - Position 3

position3 actual	before	plus Dre
A	3	12
T	4	19
G	10	32
C	7	19
position3 expected	before	plus Dre
A	3.396226	11.60377
T	5.207547	17.79245
G	9.509434	32.49057
C	5.886792	20.11321
	twosided	
P value	0.86694	
ChiStat	0.726559	
Degrees	3	

Chi Square table - Position 6

position6 actual	before	plus Dre
A	5	14
T	4	23
G	12	26
C	3	19
position6 expected	before	plus Dre
A	4.30189	14.69811
T	6.11321	20.88679
G	8.60377	29.39623
C	4.98113	17.01887
	twosided	
P value	0.279	
ChiStat	3.8423	
Degrees	3	

Chi Square table - Position 7

position7 actual	before	plus Dre
A	6	19
T	10	12
G	5	40
C	3	11
position7 expected	before	plus Dre
A	5.660377	19.33962
T	4.981132	17.01887
G	10.18868	34.81132
C	3.169811	10.83019
	twosided	
P value	0.018644	
ChiStat	9.990819	
Degrees	3	
