# Supplementary material for: Novel Heterotypic Rox Sites for Combinatorial Dre Recombination Strategies
Source: G3 (Bethesda). 2015 Dec 29;6(3):559–71. doi: 10.1534/g3.115.025841 (PMC4777119; doi:10.1534/g3.115.025841)
Supplement: Supporting Information [file supp_g3.115.025841_TableS2.pdf]

Table S2.

a, b, Medians, Means, Standard deviations, number of images, individual cells quantified, and independent transfections for experiments described in Figure 2 (a) and Figure 3 (b) .
c, d, Significance tests for the same experiments. Significance levels reported in figures reflect the outcome of the Kolmogorov-Smirnov 2 test for comparing two samples with unknown distribution.

a

HEK293-Dre transfection	Recombined/Transfected cells (%)				
	proxP-R-roxP-G	prox12-R-rox12-G	proxP-R-rox12-G	prox85-R-roxP-85	proxP-R-rox85-G
Medians	100.00	96.71	0.00	90.70	0.00
Means	99.50	94.81	0.00	90.75	0.00
Standard deviations	1.20	5.15	0.00	5.80	0.00
Images quantified	6	6	6	6	6
Independent transfections	2	2	2	2	2
Cells Counted	274	586	316	379	313

b

FREX Experiment HEK293-Dre and HEK293-Cre transfection	Recombined/Transfected cells (%)					
	pAAV-FREX		proxP-R-rox12-G		pAAV-PTPY*	
	Dre	Cre	Dre	Cre	Dre	Cre
Medians	97.92	0.00	95.67	0.00	0.00	6.46
Means	95.81	0.00	95.34	0.00	0.00	8.96
Standard deviations	5.67	0.00	1.91	0.00	0.00	5.38
Images quantified	6	6	6	6	6	6
Independent transfections	2	2	2	2	2	2
Cells Counted	112	223	436	328	3049	3042

*note = recombined cells are reported as % from overall DAPI cells, as there is no reporter expression before recombination

c

HEK293-Dre transfection	prox12-R-rox12-G vs proxP-R-rox12-G	prox85-R-roxP-85 vs proxP-R-rox85-G	proxP-R-roxP-G vs prox12-R-rox12-G	proxP-R-roxP-G vs prox85-R-roxP-85
Student T test null rejected	1	1	0	1
Student T test p value	6.93E-13	3.48E-12	0.0547	0.0047
Kolmogorov-Smirnov test null rejected	1	1	0	1
Kolmogorov-Smirnov test p value	0.0013	0.0013	0.0766	0.0122

d

FREX experiment HEK293-Dre vs Cre	Dre vs Cre pAAV-FREX	Dre vs Cre proxP-R-roxP-G vs	Dre: pAAV-FREX vs prox12-R-rox12-G	Dre vs Cre pAAV-PTPY
Student T test null rejected	1	1	0	1
Student T test p value	1.62E-12	3.35E-17	0.8487	0.0022
Kolmogorov-Smirnov test null rejected	1	1	0	1
Kolmogorov-Smirnov test p value	0.0013	0.0013	0.3180	0.0013
