# Supplementary material for: Novel Heterotypic Rox Sites for Combinatorial Dre Recombination Strategies
Source: G3 (Bethesda). 2015 Dec 29;6(3):559–71. doi: 10.1534/g3.115.025841 (PMC4777119; doi:10.1534/g3.115.025841)
Supplement: Supporting Information [file supp_g3.115.025841_FileS1.pdf]

File S1. Supplementary Methods:

Molecular modeling

The sequence of the tyrosine recombinase from Enterobacteria phage D6, Dre, was retrieved from the UniProtKB database (http://www.uniprot.org/uniprot/Q5QBE8). The Dre structure was built using the automated protein-homology modeling software incorporated in the molecular visualization, modeling, and dynamics program YASARA (KRIEGER *et al.* 2004; KRIEGER *et al.* 2006) based on the crystal structure of Cre recombinase complexed with DNA in a site-specific recombination synapse as a structural template (GUO *et al.* 1997). Atomic coordinates for the template were obtained from the protein structure database (http://www.rcsb.org/pdb), PDB file: 1CRX (2.4Å). Two protein structures were generated for the Dre recombinase complexed with a double strand DNA corresponding to either the loxP or the roxP site, respectively. Structural models were refined with the molecular dynamics package incorporated in the Yasara program using the Amber03 force field (DUAN *et al.* 2003). Complexes were neutralized with Cl^- molecules and hydrated with 12,673 water molecules in a rectangular cell 121 x 121 x 121 $Å^3$. Finally, structures of both complexes in water were equilibrated at pH7.4 and room temperature for 1 ns using periodic boundary conditions. Molecular graphics and analyses of the simulation and verification of DNA sequences were performed using an extensible program for interactive visualization and analysis of molecular structures - UCSF Chimera (PETTERSEN *et al.* 2004), developed by the Resource for Biocomputing, Visualization, and Informatics at the University of California, San Francisco (supported by NIGMS P41-GM103311).

χ^2 Script for Matlab

```
function ChiSquareMultiCathegory

% input here the desired position
position = 4

% this is a function determining the Chi Square test for cathegorical
% measurements and comparing a treatment and control distribution.
% Treatments to be compared should be columns; cathegories should be rows
% this script has to be run for each nucleotide position

[filename, pathname]= uigetfile('*.mat','Select the data you need')
load([pathname, filename]);


% combine the actual values for a given position nucleotides
positionXActual(:,1) = rox2N(:,position)
positionXActual(:,2) = rox2NxDre(:,position)
chi2stat = 0
% calculate expected tables, and chisquare differences

for i = 1:size(positionXActual,1)

    for j = 1:size(positionXActual,2)
        positionXExpected(i,j) = sum(positionXActual(i,:),2)*...
            sum(positionXActual(:,j))/sum(sum(positionXActual,1),2)
      chi2stat = chi2stat + ((positionXActual(i,j)-...
          positionXExpected(i,j))^2/positionXExpected(i,j))

    end

end
% degrees of freedom are calculated as product of (NrColumn-1)*(NrRows-1)
DegFreedom = (size(positionXActual,1)-1)*(size(positionXActual,2)-1)
p = 1 - chi2cdf(chi2stat,3)


 end
```
